# Poly(vinyl chloride) Composites with Raspberry Pomace Filler

**DOI:** 10.3390/polym13071079

**Published:** 2021-03-29

**Authors:** Jacek Mirowski, Rafał Oliwa, Mariusz Oleksy, Jolanta Tomaszewska, Joanna Ryszkowska, Grzegorz Budzik

**Affiliations:** 1 Faculty of Technology and Chemical Engineering, The J. and J. Śniadeccy University of Science and Technology in Bydgoszcz, PL-85796 Bydgoszcz, Poland; jacek.mirowski@utp.edu.pl (J.M.); jolanta.tomaszewska@utp.edu.pl (J.T.); 2 Faculty of Chemistry, Department of Polymer Composites, Rzeszow University of Technology, PL-35959 Rzeszów, Poland; molek@prz.edu.pl; 3 Faculty of Materials Science and Engineering, Warsaw University of Technology, PL-02507 Warsaw, Poland; joanna.ryszkowska@pw.edu.pl; 4 Faculty of Mechanical Engineering and Aeronautics, Department of Mechanical Engineering, Rzeszow University of Technology, PL-35959 Rzeszów, Poland; gbudzik@prz.edu.pl

**Keywords:** PVC, natural particles, plant materials, thermal stability, flame resistance, polymer composite

## Abstract

This study examined, the effect of chemically extracted raspberry pomace on the thermal stability, mechanical properties, flammability, chemical structure and processing of poly(vinyl chloride). It was observed that the pomace in this study was used to extract naphtha, thereby permitting the removal of bio-oil as a factor preventing the obtaining of homogeneous composites. Furthermore, adding 20% raspberry pomace filler after extraction extended the thermal stability time for the composites by about 30%. It was observed that composite density, impact strength, and tensile strength values decreased significantly with increasing concentrations of filler in the PVC matrix. At the same time, their modulus of elasticity and Shore hardness increased. All tested composites were characterized by a good burning resistance with a flammability rating of V0 according to the UL94 test. Adding 20 to 40% of a natural filler to the PVC matrix made it possible to obtain composites for the production of flame resistant elements that emitted less hydrogen chloride under fire conditions while ensuring good rigidity.

## 1. Introduction

Composite materials consist of at least two different components: a solid phase designated matrix, and a dispersed phase, also termed filling or reinforcement. The properties of the composites depend on the characteristics of each phase, their amount in the total volume of composites, the manner of the dispersed phase distribution in the matrix, and its geometric features. Composites are characterized by high mechanical and strength parameters at low specific gravity. Agricultural waste can also be the dispersed phase in polymer composites, and primary and recycled materials may form the matrix. Agrowaste is the most important and richest source of underexploited lignocellulose materials like seeds, hulls or husks, peels, leaves, phloem, stems, reeds, and grasses [1,2]. It may also include residue from agrofood processing: pomace (raspberry, strawberry, apple, chokeberry, or currant), corn meal, straw, oat and rice husks, plum and cherry stones, sugarcane, almond shells, wheat husks, lime and nut leaves, peanut, hazelnut and pistachio shells, and sunflower husks [3,4,5,6]. During their decomposition, greenhouse gases are released, so for economic and environmental reasons agrowaste is desired for the production of polymer composites. The use of agrowaste makes possible polymer composites with excellent properties such as improved mechanical strength, barriers to water and oxygen, high dimensional stability, and thermal stability. [7,8]. Agrowaste and thermoplastic composites are used in the automobile and construction industries and have a variety of consumer applications due to their low cost [9,10]. Moreover, they attract the interest of many researchers [1,11,12,13]. In addition to the many advantages associated with the use of these fillers, the preparation of thermoplastic composites with agrowaste is associated with difficulties, because, like other lignocellulose materials, it consists of primary ingredients, namely, cellulose, hemicellulose, and lignin, and secondary ingredients that include proteins, waxes, fats, pectins, resins, tannins, dyes and mineral salts. The properties of agrowaste result from the chemical constitution and structure of both primary and secondary ingredients [14,15,16,17,18]. Adding agrowaste to a polymer involves bringing in primary and secondary ingredients as well as the water and air contained in their particles. These ingredients may significantly affect the melt mixing rheology and properties of the composites produced. One of the methods to improve the processing properties of PVC composites is to improve the compatibility of natural fillers with the PVC matrix by modifying their surface using a coupling agent [19,20] or adding fillers [21] and nanofillers [22].

Agrowaste has been explored worldwide as a natural and cost-effective filler for enhancing the performance properties of PVC composites. The subject of research shown in this article includes composites based on poly(vinyl chloride)(PVC) with fillers of raspberry processing residues. However, proper preparation of the filler is a prerequisite for obtaining composite material for further forming. For this purpose, the chemical extraction process of raspberry pomace was carried out and composites containing 20–40% wt. of natural filler were prepared. The influence of the content of modified raspberry pomace on the mechanical properties, thermal stability and flame resistance of obtained PVC composites were investigated.

## 2. Materials and Methods

### 2.1. Raw Materials for Composite Materials Production

The composite matrix consisted of unplasticized PVC S61 dry blend (Anwil S.A., Włocławek, Poland). The PVC dry blend is shown in Table 1.

Production residue of raspberry pomace (RR) from Agropol Sp. z o.o. Ltd. in Góra Kalwaria, Poland, was used as the filler. The RR pomace was ground in a burr grinder, and then dried at 105 °C for 4 h (RRGD). The pomace was then subjected to the Soxhlet extraction process using extraction naphtha (RRGED) and then held for 4 h at 105 °C to evaporate the solvent residue.

Prepared fillers and PVC were used to make the composites. Their composition is compared in Table 2.

### 2.2. Preparation of Samples for Tests

Poly(vinyl chloride) composites with raspberry pomace were obtained by mixing in a molten state with the FDO 234H type Brabender plastograph (Brabender GmbH & Co. KG, Duisburg, Germany). The kneading of PVC mixes with different filler content was carried out in a chamber with a wall temperature of 185 °C under a main rotor rotational speed of 30 min^−1^ at friction 1:1.5. The kneading time was 15 minutes. The unfilled PVC mix was processed under the same conditions. After chilling, the plasticized materials were ground in a milling grinder of the authors’ own design, and the resulting ground material was moulded at 190 °C for 6 minutes under a maximum working pressure of 170 bar. The obtained 2- and 4-mm thick moulded pieces were used to cut out (mill) specimens for mechanical (tensile) testing and beams for impact, oxygen index and flammability tests.

### 2.3. Testing Method

#### 2.3.1. Plastograph Analysis

Homogeneous materials for further tests were obtained as a result of processing polymer mixes by kneading in the Brabender plastograph chamber (Brabender GmbH & Co. KG, Duisburg, Germany). Moreover, the recording of rotor torque as a function of time allowed observation of the change dynamics occurring in the processed PVC and composite materials according to the so-called gelation process [23,24,25,26,27,28].

In a closed, heated chamber, the mix was moved and kneaded by two horizontal profiled rotors. The following parameters were recorded during the test: the actual temperature of the kneaded mix and the torque of rotors as a function of time in the form of a diagram called the plastogram.

**Figure 1:**
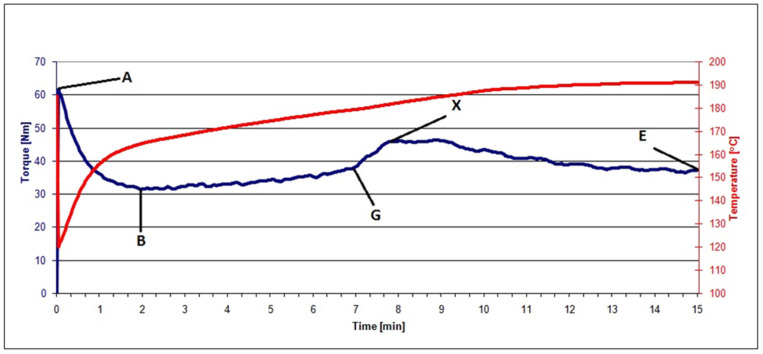
An illustration of a plastogram for kneading of PVC mix containing 40% of raspberry pomace after extraction (RRGED).

Figure 1 is an illustration of a plastogram for the kneading of PVC mix containing 40% raspberry pomace after extraction (RRGED) with marked characteristic values bound with the PVC gelation progress.

Rapid-torque increase to the maximum in point A is the effect of material loading into the chamber; at the same time, the temperature measured inside the chamber dropped. Then, the torque decreased to its minimum at point B, passed inflexion point G, and reached its maximum at point X. Such a curve course at this stage indicated particle coalescence [29]. At the same time, the temperature of the processed PVC slowly increased until the set value was reached; most often, it increased further near the points G–X. After passing point X, the torque slowly dropped and then stabilized (point E), which indicated that all ingredients were mixed. In turn, the material temperature still slowly increased between points X and E. The measurements were most often carried out until the torque value stabilized. This study presented an analysis of the following values read out from plastograms: maximum torque at the gel point (M_X_), actual temperature at the gel point (T_X_), time to reach maximum torque (t_X_), torque (M_E_) and actual mix temperature (T_E_) at the end point [23,24,25,26].

#### 2.3.2. Thermal Stability

Thermogravimetric analysis and the Congo red test were used to investigate the thermal stability of the samples of filler and composites with a PVC matrix. Congo red thermal stability was determined at 200 °C in accordance with the PN-EN ISO 306:2006 standard.

Thermogravimetric analysis (TGA) for samples of raspberry pomace fillers and PVC-based composites containing them was carried out using the TGA Q500 (TA Instrument, New Castle, DE, USA). The 10 ± 1 mg samples were tested in a nitrogen atmosphere and heated at the rate of 10 °C/min from room temperature to 950 °C. Mass accuracy and temperature precision of device were 0.5% and 0.1 °C, respectively. The results were analysed using the Universal Analysis 2000 version 4.7A application from TA Instruments (New Castle, DE, USA).

#### 2.3.3. Chemical Structure

The chemical structure of the samples of fillers and PVC composites containing them was characterized on the basis of the analysis of absorption spectra obtained using the FTIR Nicolet 6700 spectrophotometer (Thermo Electron Corporation, Waltham, MA, USA) with the ATR attachment (suppressed total reflection). Two samples from each part were scanned 64 times in the range of wave number 4000–400 cm^−1^. The obtained spectra analysis was carried out using the OMNIC 8.2.0.387 application from Thermo Fisher Scientific Inc. (Waltham, MA, USA).

#### 2.3.4. Thermal Analysis

Thermal analysis of raspberry pomace was carried out using differential scanning calorimetry (DSC) with the DSC Q1000 of TA Instrument (New Castle, DE, USA). The measurements were carried out in a helium atmosphere in hermetic aluminium crucibles. Approx. 6 mg specimens were heated at a rate of 10 °C min^−1^ in a temperature range from −80 °C to 150 °C. Temperature accuracy of device is 0.1 °C.

#### 2.3.5. Mass Loss

Raspberry pomace mass loss was analysed using the MA 50/1.R moisture balance produced by Radwag (Radom, Poland) at the temperature of 105 °C for 10 h.

#### 2.3.6. Density Determination

The density of PVC composites with raspberry pomace was determined using the Pycnomatic helium pycnometer from Thermo Fisher Scientific (Waltham, MA, USA), according to PN-EN ISO 1183-3. Five specimens from each composite type were tested.

#### 2.3.7. Mechanical Properties in Tensile Testing

Mechanical properties of the tensile testing of PVC composites with raspberry pomace were determined in accordance with the ISO 527-1 standard using samples in the form of 1 BA-type dumbbells. The INSTRON 5967 testing machine (Instron, Norwood, MA, USA) was used and controlled by the Bluehill 3 application and equipped with a video extensometer. The samples were tensioned at the 1 mm min^−1^ to 0.25% strain and then at 50 mm min^−1^. The results are the arithmetic means of 10 tests for each type of composite.

#### 2.3.8. Charpy Impact Strength

Charpy impact strength for the composite samples in the form of bars sized 100 × 10 × 4 mm was determined in accordance with PN-EN ISO 179-1 using a hammer with an impact energy of 1 J from Ceast/Instron (Instron, Norwood, MA, USA). The arithmetic means of 10 measurements was taken as the final result.

#### 2.3.9. Shore Hardness

The hardness of the composites was determined using the Shore D method according to PN-EN ISO 868. The measurements were performed for ten specimens of each composite type, using Shore D 856.006 apparatus (Hildebrand Prüf und Meßtechnik GmbH, Wendlingen a. N., Germany).

#### 2.3.10. Oxygen Index Determination

Oxygen index tests for the 10 samples of each type of composite were carried out in accordance with the EN ISO 4589-2 standard using an instrument of Fire Testing Technology Ltd. (East Grinstead, UK). Samples in the form of bars sized 100 mm × 10 mm × 4 mm were used for testing. The sample was placed vertically in a holder in the middle of a glass chimney, and then the top of the shaped piece was ignited with a gas burner. A mixture of nitrogen and oxygen flew through the chimney, and the oxygen content was changed every 0.1%. The purpose of the test was to determine the minimum oxygen concentration in the flowing mixture, for which the permanent sample burning was observed.

#### 2.3.11. Flammability—Test UL94

A flammability test for PVC/RRGED composites was carried out in the UL94 test chamber (produced by FTT Ltd., East Grinstead, UK) according to PN-EN 60695-11-10. The measurements were made with the sample beam in a vertical position and a methane fed burner at a height of 20 mm. The specimens were 127 mm long, 12.7 mm wide, and 4 mm thick. A burner was placed under the sample for 10 s during the test, and the burning time t_1_ was measured after burner removal. After the flame went out, the burner was brought back for another 10 s and the burning time t_2_ was measured until the shaped piece stopped burning again. Each type of test was performed on at least three samples from each series.

## 3. Results and Discussion

### 3.1. Filler Testing Results

**Figure 2:**
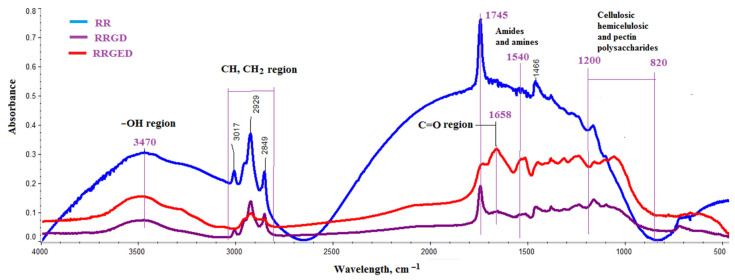
FTIR analysis results for raspberry pomace: in the initial state (RR), after grinding and drying (RRGD), and after extraction and heating (RRGED).

**Figure 3:**
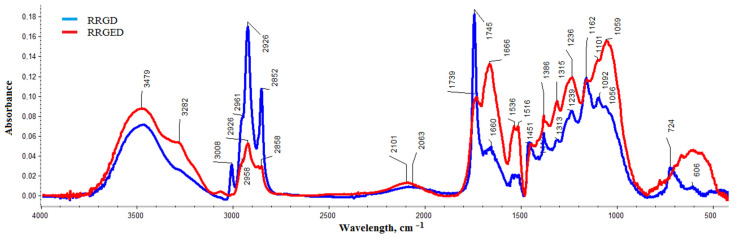
Comparison of raspberry pomace spectra before (RRGD) and after extraction (RRGED).

Raspberry pomace used as a composite filler in the form delivered by the producer (RR) was examined using ATR–FTIR spectroscopy after grinding and drying (RRGD), and after grinding, extraction and drying (RRGED). Figure 2 and Figure 3 shows a representative spectrum for each type of sample. The bands appearing in the FTIR spectrum of the analysed materials are alike (Figure 2). The most intense absorption band centred at ≈3470 cm^−1^, corresponding to water [30], occurred in the RR spectrum, and is much weaker in the RRGD and RRGED spectra. These absorption bands appearing between 3770 and 3030 cm^−1^ were attributed to the O−H stretching vibrations as well, indicating the existence of phenols, alcohols, or carboxylic acids.

The sharp peak at 2929 cm^−1^ and the side weaker peak 2849 cm^−1^ corresponded to asymmetric and symmetric CH_2_ bonds [31]. These bands are intense in the RR spectrum, but in the RRGD and RRGED spectra their intensity was much smaller. The vibrations between 2800 and 3000 cm^−1^ are consistent with the presence of long-chain fatty acids, waxes, carotenoids, and phytosterols [32,33]. For all samples, the stretching bands occurred in the spectrum in the 2900–3030 cm^−1^ region and originated from vibrations of the C-H group. The band 3017 cm^−1^ does not appear in the RRGED spectrum. The absorption band between 1600 and 1800 cm^−1^ (peaks at 1745 and 1600 cm^−1^) indicated the presence of C=O in either a carboxylic acid/ester or aldehyde/ketone group [34,35].

Strong bands due to carbonyl groups can also be seen in the region 1710–1745 cm^−1^. In Figure 2, strong bands of the C=O stretching vibration of ester bonds at 1745 cm^−1^ are visible in the RR and RRGD spectra. The lignin aromatic ring stretch band should appear in the aromatic ring stretch at 1604 cm^−1^ with an accompanying band at 1658 cm^−1^ [30]. No bands from the aromatic ring appeared in the RR and RRGD spectra. In RRGED, there was a band at 1658 cm^−1^ which may have been associated with the aromatic ring stretch, and a band at 1540 cm^−1^ resulting from vibrations of the N–H in–plane bending of amide and amine groups. A weak band at 1413 cm^−1^ also confirmed the C–H groups in alkanes. The peaks at 1413 and 1239–1236 cm^−1^ are likely the result of the aromatic −O, O−O−C, and C−N bonds. The most intense peak at 1060 cm^−1^ is an indicator of C−O or C−C stretching [36].

The typical infrared pattern of lignocellulosic materials is observed in the region 900–1200 cm^−1^ [30,31,37].

After the grinding and drying (RRGD) and after grinding, extraction, and drying (RRGED) raspberry pomace spectra were compared to evaluate the extraction efficiency (Figure 3). Spectra analysis results are shown in Table 3 along with the direction of changes in individual bands in RRGED compared to RRGD.

To determine which compounds were removed during RR preparation for composite production, the table also showed the analysis results of the raspberry pomace derivative oil spectrum [38]. It was noted that most bands that did not appear on the RRGED spectrum, or whose intensity was distinctly smaller compared to the RRGD spectrum, were those that appeared in the spectrum of oil obtained from RR.

**Figure 4:**
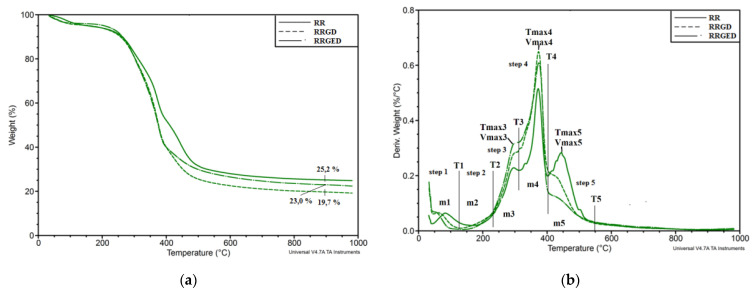
Thermograms of different raspberry pomace forms: (**a**) TG, (**b**) DTG, with description of specified parameters.

Mass change curves (TG) allowed it to be observed that significant differences among the degradation trajectories of RR, RRGD and RRGED appeared within the range of 250 to 450 °C (Figure 4a). After the degradation process at 900 °C, the greatest volume of ash remained after the degradation of RR, and the least volume after the degradation of RRGD. Mass change derivative curves (DTG) showed five main degradation stages (Figure 4b), which are described and compared in Table 4.

The first stage of thermal degradation connected with the evaporation of water molecules, and water bonded by hydrogen bonds ended at temperatures of 168 °C (RR), 127 °C (RRGD), and 139 °C (RRGED) (Table 4). During this stage 4.0–5.3% of the sample mass was lost. The second stage, which ended at 231–235 °C and involved a mass loss reaching 1.9–3.2% indicated the removal of a strongly bonded water molecule and decomposition of the weaker hydroxyl bonds. The third stage ended at 309–318 °C, and 14.0–20.5% of the mass was lost, mostly in RR. The third stage (230–320 °C) may result from the degradation of hemicellulosis [39]. Dorez et al. [40] linked this range with depolymerisation of hemicellulose and the cleavage of glycosidic linkages of cellulose. Asim et al. [41] linked this stage of degradation with the thermal decomposition of hemicellulose, lignin, pectin, and glycosidic linkages of cellulose, whereas Essabir et al. [42], analysed the process of coir residues decomposition within the range of 270–330 °C and observed that it was associated with the degradation of hemicelluloses and pectins. The fourth stage ranging from 320 to 400 °C led to a minor mass loss in the RR and about twice as much loss for RRGD and RRGED. According to Dorez et al. [40], the process within the 340–370 °C range was assigned to the degradation of α-cellulose, and according to Assim et al. [41] the degradation occurred within the range of 389 to 449 °C. During the fifth stage (400–550 °C) the analysed residues showed a loss of 23.3% from RR and 16.6% and 12.1% of mass from RRGD and RRGED, respectively. According to Essabir et al. [42], the degradation of cellulose and lignin appears in this range. A like interpretation was shown by Assim et al. [43] and Methacanonet et al. [44].

Bio-oils are formed during the extraction of coir residues, and their degradation occurs within temperatures of 130 to 450 °C [45]. The oil degradation process overlaps with the degradation processes of primary ingredients, which build food residues. In the third stage, the degradation rate was highest for oil-free pomace (RRGED). In all probability, the extraction process triggered a change in the RRGED structure that enhanced the degradation rate of this material. Active hydrogen was formed during the extraction carried out with ethanol, and free radicals [46] break the C–C and C–O bonds in cellulose molecules to form sorbitol, which converts to alkanes and alkenes [47]. These processes foster thermal degradation of cellulose, which explains the greater mass loss in RRGED at stage 3 and the simultaneous increase in the maximum degradation rate compared to RRGD.

During the succeeding stages, the RRGED degradation rate is lower compared to RR and RRGD, which results from oil mass loss. The oil degradation rate is greater than the degradation rate of the primary ingredients in lignocellulose residues.

The most significant weight loss in the investigated food residue was shown by a sharp trough between about 320 and 400 °C, with a maximum mass loss rate within the range of 0.52 to 0.65 wt.%/°C. Much the same results were received in the study by Opatokun et al. [48].

**Figure 5:**
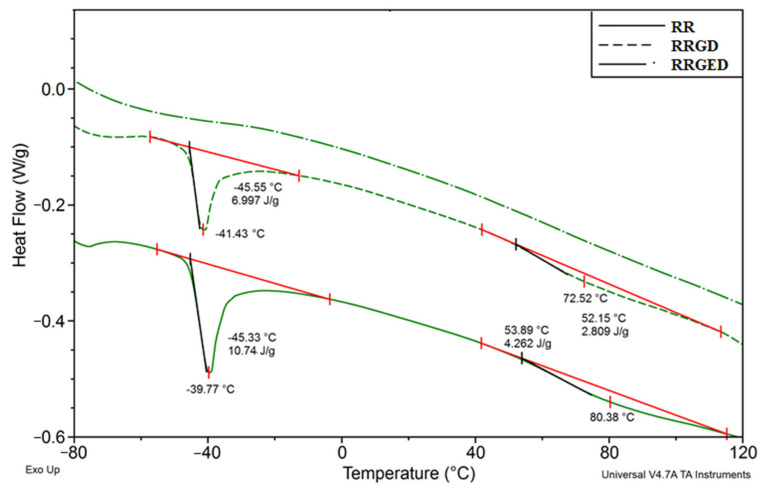
DSC thermograms of different forms of raspberry residues.

The DSC measurements of all residues were carried out by heating the samples from −80 to 120 °C as presented in Figure 5. The representative DCS thermograms of the RR and RRGD residues showed the occurrence of an endothermic peak with the minimum at approx. –40 °C. This peak may be related to the melting of crystallites commonly associated with methyl esters of unsaturated fatty acids [49]. The DSC thermogram of RRGED show no peaks present in RR and RRGD, which confirms that bio-oil was removed during the extraction. The second peak indicated the loss of water molecules at the lower temperatures 80 and 72 °C for RR and RRGD, respectively.

**Figure 6:**
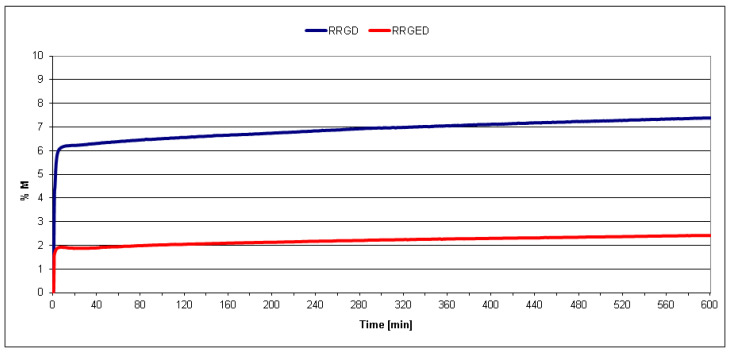
Change in the mass of raspberry waste samples before and after the extraction process carried out using extraction naphtha (RRGD and RRGED, respectively).

The efficiency of the modification of RRGD pomaces by naphtha extraction was additionally assessed using the moisture balance. The analysis of mass loss curves for the samples in a function of heating time (Figure 6) showed that about 7.4% of raspberry pomace mass was lost after grinding and drying and before extraction (RRGD) by heating for 10 hours, while only around 2.4% of the mass was lost from the samples that underwent additional extraction and heating. It can be assumed that the 5% higher mass loss for the RRGD sample during heating in a moisture balance was connected with the loss of bio-oil, which degraded during prolonged heating and gradually released gaseous products. This effect was not observed in the case of RRGED because bio-oil had been effectively extracted before, as confirmed by the thermal analysis carried out using the DSC method.

### 3.2. Composite Testing Results

PVC composites with previously ground and dried fillers, specifically PVC/20 RRGD, PVC/30 RRGD, PVC/40 RRGD, were porous after removal from the Brabender chamber. The probable reason for this was the extraction of bio-oil during processing; the oil content in the filler assessed on the basis of the mass loss check in the TGA analysis was 5%. As a result, these composites were useless for making further test samples.

**Figure 7:**
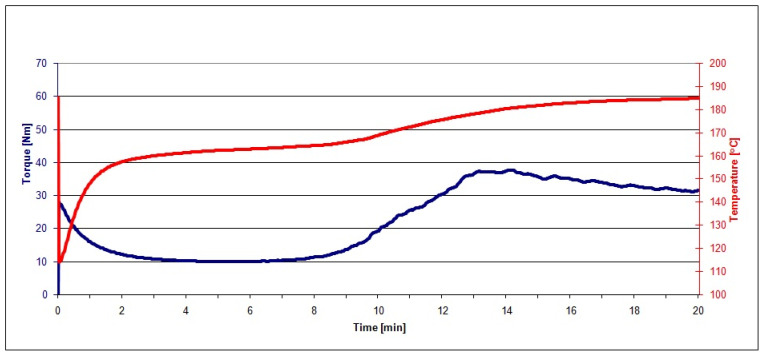
Kneading process for the PVC mix.

**Figure 8:**
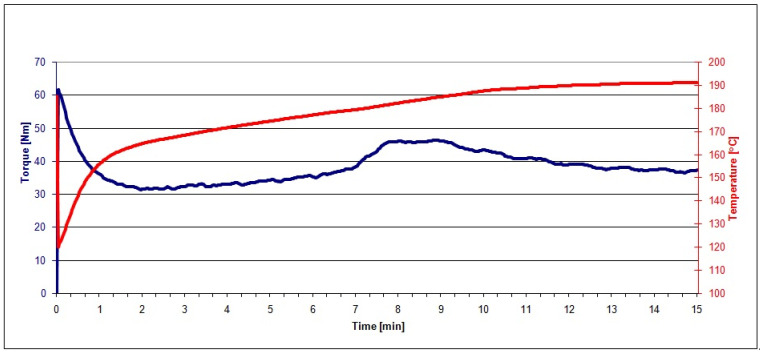
Kneading process for the PVC/40 RRGED mix.

The PVC mix and the mixes of PVC and raspberry pomace subjected to grinding, extraction, and heating (PVC/20 RRGED, PVC/30 RRGED, PVC/40 RRGED) after plasticisation in the Brabender chamber were visually homogeneous. The correctness of the kneading process was confirmed by the progress of plastograms characteristic for PVC and composites based on it (Figure 7 and Figure 8).

The characteristic values of torque, actual temperature of processed mixes, and time (see Figure 1) read out from the plastograms of PVC composites with filler (RRGED) are compared in Table 5.

From the plastograph analysis results, it was noted that the dried and extracted raspberry pomace that was added to the unplasticized PVC mix increased the value of maximum torque at the gelation point and at the kneading end point, while M_X_ and M_E_ values grew with increasing RRGED filler content in the polymer material. However, the maximum torque for mixes with 40% filler content does not exceed 50 Nm. A similar dependence was observed in the case of the actual temperature of the kneaded mix at the gelation point (T_X_) and at the end point (T_E_). At the same time, a considerable reduction of the time needed to reach the maximum torque (t_X_) was confirmed compared to the gelation time of the unfilled dry blend. Time t_X_ about 5–6 min shorter regardless of the RRGED content in the PVC composite, which is economically advantageous for PVC-composite processing.

Gelation of PVC in processing conditions, including kneading, depends on the applied shear rate, temperature and PVC composition [23,24,25,26]. The action of shear stress causes the disintegration of primary grains into smaller elements, i.e., agglomerates of primary particles. Subsequently, heating causes the progressive plasticization and melting of these elements. These thermal–mechanical changes to the morphological structure of PVC grains also involve the smallest elements in the grains, the so-called primary crystallites [23,24,25,26]. It may be assumed that the addition of a filler promotes the release of more heat in kneaded compounds, as a result of the friction of filler particles against one another or against PVC grains, of PVC grains against one another, or of filler particles or PVC grains against the surface of the chamber wall. Moreover, for the same batch weight, the volume of the PVC/RRGED mixture is larger than that of the PVC, which results in higher shear stress in the plastograph chamber. Due to these effects, the gelation of PVC/RRGED mixtures is faster than that of PVC; similar effects were found in case of PVC with wood flour [28].

The plastograph analysis results, in particular the increase of torque during kneading, confirmed that the filler added to the mixes did not contain substances that would induce plasticising and lubricating effects.

**Figure 9:**
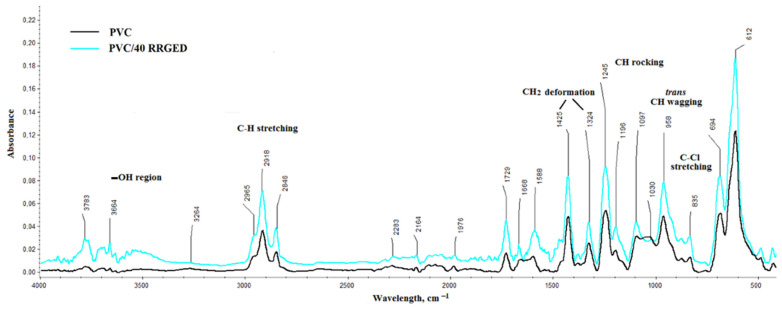
The FTIR spectra of the PVC composites with raspberry residues after grinding, drying, and extraction process.

Figure 9 shows the FTIR spectra of PVC-based composites with a description of characteristic bands. The bands typical for poly(vinyl chloride) are observed on the FTIR spectra of PVC composites with 40% RRGED filler content (Figure 9). The absorption peak observed at 2918 cm^−1^ can be assigned to C–H stretching vibrations. The bands at 1324 cm^−1^ and 1425 cm^−1^ can be attributed to the deformation C–H of CHCl and CH_2_ deformation wagging, respectively. The peak at 958 cm^−1^ corresponded to trans C–H wagging vibration and CH_2_ rocking, and the one at 1097 cm^−1^ to the stretching vibration of C–C. The band at 833 cm^−1^ was related to the stretching vibration of C–Cl. The peak at 1246 cm^−1^ was attributed to the bending vibration of C–H originating from the CHCl groups of the PVC polymer as well as to C–H rocking [50,51,52].

The bands related to filler presence are clearly visible in the spectra of PVC/RRGED composites: the intermolecular bonded O–H stretching vibration centred at 3283 cm^−1^, aromatic-skeletal vibration, C–O stretching vibration (1104 cm^−1^) and C–O wagging vibration of polysaccharides in cellulose (1095 cm^−1^).

Apart from the 3272 cm^−1^ in the spectra, bands within the range of 3536 to 3773 cm^−1^ are present (probably also from OH), and their intensity is greater in the spectrum of PVC composite with 40% filler content. This band probably originated from the vibrations of water bonded in the filler structure. Moreover, the PVC and PVC/RRGED spectra show bands O=C=O stretching (2283 cm^−1^), C=C=O stretching (2164 cm^−1^) and C–H bending in the aromatic compound (1976 cm^−1^). These bands may originate from vibrations of groups appearing in the structure of the modifiers used in the PVC mix.

Material grounds after kneading in the Brabender plastograph chamber were used to determine static thermal stability using the Congo red method. The analysis of the results compared in Table 6 shows that after adding 20% of RRGED filler to the polymer matrix, PVC thermal stability time increased from 22 to 29 min. An increase of filler concentration to 30 and 40% extended by 1–2 min. the time after which hydrogen chloride was released. Regarding the processing technology, the improvement of the thermal stability of PVC composites after adding the filler is an extremely advantageous property of the obtained materials. The thermal stability of PVC/RRGED composites that increased with the growth of filler content on the one hand was connected with the dropping polymer content in the analysed material, and on the other hand with the thermal conduction of the material modified after adding the filler.

**Figure 10:**
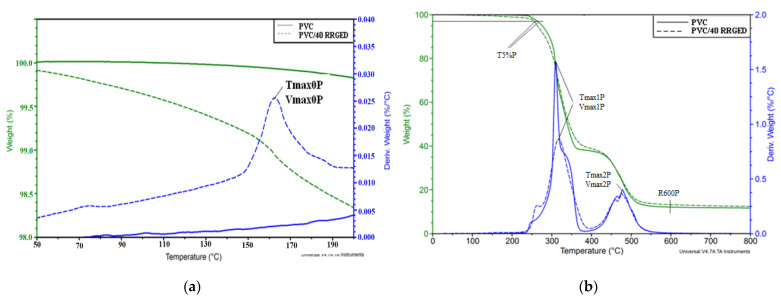
The TGA thermograms of poly(vinyl chloride and composites with 40% content of raspberry pomace after extraction: (**a**) The results of TGA analysis to 200 °C; (**b**) The results of TGA analysis.

Thermogravimetric analysis of PVC and PVC/RRGED composites was carried out to determine the size of thermal transitions connected with thermal degradation and to the temperatures at which these transitions occurred. Figure 10 shows thermograms of PVC and composites containing 40% filler from 50 to 200 °C and within the full range of 30 to 800 °C, while the analysis results of mass change curves (TG) and mass change derivative curves (DTG) for all analysed materials are compared in Table 6. The TG curves allowed for the finding of the temperature for 5% mass loss (T_5%P_) and for residue after degradation at 600 °C (R_600P_), and the DTG curves were used to determine the temperature for the maximum degradation rate at each stage (T_max0P_, T_max1P_, T_max2P_), for maximum degradation rate at the following temperatures (V_max0P_, V_max1P_, V_max2P_), and for mass change during each of the stages ∆m_0P_, ∆m_1P_, ∆m_2P_, respectively.

The temperature range connected with the beginning of degradation is important from the point of view of processing PVC-based composites; therefore, Figure 10a shows an example thermogram for PVC and PVC composite with 40% content of the RRGDE filler in the range of 50 to 200 °C. A thermogram of the PVC/RRGED composite allowed observation of the occurrence of the peak within 150–190 °C connected with the loss of water bonded with hydrogen bonds in the filler structure (Figure 10a).

An analysis of the data read out from the TG and DTG curves for PVC and composites containing 20, 30, and 40% of filler indicates that the rate of water loss and its volume increases with the increasing of the volume of filler in the matrix. It should be added, however, that the mass loss for the composite with the greatest filler content, 40% at 165 °C, is only 1.1%.

Degradation of PVC and PVC-based composites containing 40% of the RRGED filler proceeds in two stages. The first degradation stage observed within the range of 230–380 °C is assigned to the progressive PVC dehydrochlorination and the formation of conjugated polyene structure [53,54,55]. At this stage, the composite mass loss decreases with increasing concentration of filler in the matrix and ranges from 59.5% to 54.9%. It is smaller compared to the 61.4% mass loss observed for PVC that corresponds to the value specified in the literature for unmodified polymers [56]. The degradation rate at this stage decreased with increasing RRGED content in the polymer matrix (Table 6b). Moreover, analysis of the thermogram in Figure 10b and the data in Table 6b indicated that the temperature for 5% mass loss decreased with increasing filler volume in the PVC matrix. Therefore, the degradation of composites in this temperature range proceeded more intensely than for the unfilled PVC, probably due to the release of thermally unstable organic filler ingredients. The second stage of PVC decomposition in the range of 430 to 600 °C involved the decomposition of the formed cross-linked polyene structure, which resulted in residual char [57]. At this stage, the degradation rate of the PVC/RRGED composites is slightly lower than for the unfilled PVC, and the mass loss was also smaller. This effect proved that the added filler reduced the emissions of gaseous decomposition products, thus triggering an increase in ash residue after degradation at 600 °C, which increased with increasing RRGED concentration in the polymer matrix.

**Figure 11:**
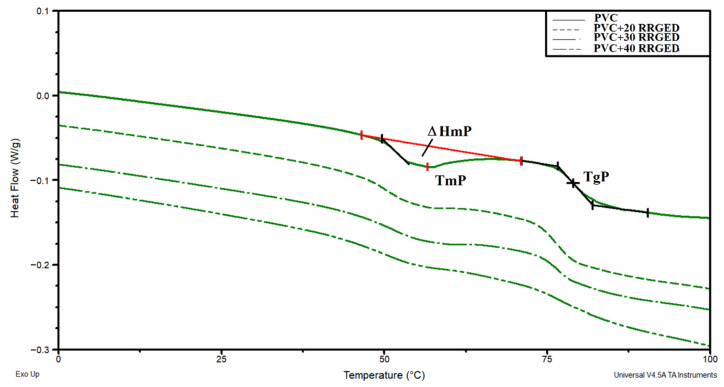
The DSC thermograms of PVC and PVC-based composites with varying content of the RRGED filler.

Completed thermal tests also included DSC analysis of the samples of PVC and PVC-based composites with varying RRGED filler content (Figure 11). Glass transition temperature determined on the basis of the DSC thermograms is 79 °C for PVC, and 76.0 to 76.7 °C for PVC-based composites, and in practice this is independent of filler concentration (Table 7). Lower glass transition temperature for the PVC/RRGED composites indicates greater mobility of PVC macroparticles. This effect results from reduced interactions between adjacent polymer chains induced by adding filler particles. The obtained results prove that the RRGED filler triggers slight PVC plasticisation [58]. Moreover, the DSC thermograms show an endothermic transition within the temperature range of 45 to 65 °C with a minimum at 54.5–56.1 °C. The enthalpy of this transition is 0.53–1.72 J g^−1^ and decreased with increasing RRGED volume in the composite. Probably, this transition was the result of the melting of an additive used in the PVC mix, namely, the external/internal lubricant Loxiol G-32 [59].

The following parameters were analysed to assess the filler impact on the physical and mechanical properties of PVC-based composites: their density (D), Shore hardness D (H), impact strength (U), and mechanical properties determined in the static-tensile test. The following were determined during mechanical tests using tensile testing: E_t_—modulus of elasticity; σ_y_, stress at yield; ε_y_ç strain at yield; σ_m_, maximum stress; ε_m_, strain at maximum stress; σ_b_, stress at break; ε_b_, strain at break. Results of these tests are compared in Table 8.

The obtained results indicate that the added filler significantly affected the mechanical properties of the PVC composites with raspberry pomace residue. The greatest changes were observed in impact strength; adding 20% by mass of the RRGED filler reduces it by 10 times compared to the reference sample. Further increasing the filler content did not result in significant changes in impact strength, which reached 3.2 kJ m^−2^ for a composite with 40% filler content. Young’s modulus increased with growing filler concentration in the composite; adding 40% of RRGED to the composite resulted in an increase in the modulus value by 32.5% compared to the sample of unfilled PVC. This change in properties was typical for most of the composite materials, which became more rigid and brittle as the filler content increased [60,61,62]. The consequence of a high filling degree of PVC composites also included reduced maximum stress and stress at break. The values of these stresses were close, which proved the lack of yield point for the PVC/RRGED composites. Yield stress was observed during the tension of unfilled PVC samples, and the corresponding stress value equalled a maximum stress of about 55 MPa. In this case, stress at break was 34 MPa and exceeded the values σ_b_ of the composites analysed in this study. The greatest drop in maximum stress and stress at break, by 61.6% and 33.3%, respectively, compared to the unmodified matrix, was observed for the composite containing 40% by mass of RRGED filler. As reported in the literature [22,63], such a decrease in tensile strength is probably related to the stress concentration around the filler, which may be attributed to the weak adhesion between the natural fillers and the polymer matrix. Adding raspberry pomace filler slightly increases the hardness of the PVC-based composites determined by using the Shore method. The hardness of the composite containing 40% of filler was only about 5% greater compared to the polymer matrix. The density of PVC/RRGED composites was lower than the PVC density and decreased with increasing filler concentration in the matrix. In the case of the composite with maximum filler concentration used for the purposes of this study, the density was around 3% lower than the matrix density.

Poly(vinyl chloride) (PVC) is one of the most widely used engineering plastics [64], and its applications are frequently determined by its high fire resistance. Pristine PVC has excellent intrinsic flame retardancy with a limiting oxygen index (LOI) of 37.4%, but its modification often reduces the LOI value [65]. Therefore, the scope of this study included evaluation of the fire resistance of the produced PVC/RRGED composites. The flammability test results are compared in Table 9.

The results compared in Table 9 allowed it to be noted that the adding of the RRGED filler reduces the limiting oxygen index values for PVC-based composites due to the organic character of the filler. Moreover, the oxygen index decreased with the increasing of the filler content. As a result, the composite containing 40% by mass of RRGED had an oxygen index of 32%, which differed 14.4% from the unmodified PVC. However, it is worth noting that despite the considerable change in LOI, all composites were flammable, with a flammability rating of V0 according to the UL94 test. This result was obtained first of all from the flammable properties of PVC as the matrix for composites. Additionally, scale forming as a result of organic filler carbonisation may work as a protective barrier inhibiting heat and mass, thus reducing the emission and combustion of gaseous decomposition products [64].

## 4. Conclusions

Raspberry pomace may be used to produce composites based on unplasticized PVC containing up to 40% filler. However, proper preparation of the filler is a prerequisite for a correct PVC gelation process, and consequently for obtaining composite material for further forming. Pomace processing was applied for the purposes of this study, which involved using chemical extraction to extract naphtha, allowed the removal of bio-oil as a factor, which was preventing the obtaining of homogeneous composites. Adding 20% of raspberry pomace filler after extraction extended the thermal stability time of the composites by around 30%. It was observed that the composite density, impact strength, and tensile strength values decreased significantly with the increasing concentration of filler in a PVC matrix. At the same time, their modulus of elasticity and Shore hardness increased. Although LOI value decreased with the increasing content of filler in the polymer matrix, all materials were characterized by a good burning resistance having a flammability rating of V0 according to the UL94 test. Adding to the PVC matrix, 20 to 40% of a natural filler after chemical extraction permits the use of the obtained composites for the production of flammable elements with reduced hydrogen chloride emission in fire conditions while ensuring good rigidity.

## Figures and Tables

**Table 1:** Composition of the PVC D-599 dry blend.

Component	Trade Name	Concentration, phr	Function
Poly(vinyl chloride)	PVC Neralit 601	100	Main component
Organotin	Patstab 2310	2	Thermal stabilizer
Calcium stearate	Ceasit I	1.2	Thermal stabilizer
Fatty acid ester	Loxiol G-32	1.5	External and internal lubricant
Acrylic-based polymer	Poraloid K-125	1	Universal lubricant
Acrylic-based polymer	Poraloid K-175	1	External lubricant
Paraffin	Naftolube FTP	0.5	External lubricant

**Table 2:** The composition of the PVC-based composites.

Sample Type	Filler	Concentration, % mas
PVC	–	–
PVC/20 RRGD	RRGD	20
PVC/30 RRGD	RRGD	30
PVC/40 RRGD	RRGD	40
PVC/20 RRGED	RRGED	20
PVC/30 RRGED	RRGED	30
PVC/40 RRGED	RRGED	40

**Table 3:** Comparison of the RRGD and RRGED FTIR spectra.

Bands	Wavelength of RRGD, cm^−1^	Wavelength of RRGED, cm^−1^	Intensity ^1^	Wavelength of RR Oil, cm^−1^ [32]
O–H stretching vibrations of water, phenols, alcohols, or carboxylic acids	3479	3479	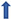	–
intermolecular bonded O–H stretching vibrations	3282	3282	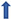	–
CH_2_ deformation bands	3008	–	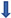	3017
asymmetric CH_3_ stretching	2958	2961	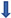	–
the stretching vibrations of aliphatic C–H in CH_2_	2926	2926	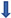	2929
the stretching vibrations of aliphatic C–H in terminal CH_3_ groups	2852	2858	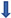	2849
C=O stretching vibration of carboxylic acids of the ester	1745	1739	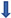	1745
The aromatic ring stretch	1660	1668	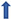	–
The vibrations of the N–H in-plane bending of amides and amines group	1536	1536	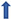	–
C–C stretching,	1516	1516	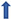	–
CH_2_ scissoring	1440	1451	=	1460
The symmetric HCH bending	1386	1386	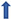	1377
CH_2_ wagging mode of cellulose	1313	1315	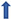	–
The aromatic −O, O−O−C, and C−N bonds	1239	1236	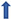	–
the C−O stretching alcohols groups C–C stretching	1162	1162	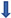	1157
polysaccharides region 900–1150 cm^−1^	1092	1100	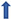	–
C–O–C asymmetric stretches for the mixed phenyl/methyl ether linkage	1056	1059	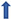	–
the aromatic compounds present in the oil	724	–	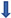	721

^1^ RRGED versus RRGD: 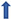 increase in intensity, 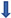 drop in intensity, = no change.

**Table 4:** Results of TGA analysis of raspberry pomace.

Parameter	RR	RRGD	RRGED
Step 1			
Stage 1 end temperature (T_1_), °C	168	127	139
Weight change (m_1_), %	5.3	4.6	4.0
Step 2			
Stage 2 end temperature (T_2_), °C	233	235	231
Weight change (m_2_), %	1.9	3.2	2.3
Step 3			
Stage 3 end temperature (T_3_), °C	315	309	318
Weight change (m_3_), %	20.5	14.0	18.9
Temperature at V_max3_ (T_max3_), °C	294	301	300
Maximum degradation rate (V_max3_), %/°C	0.23	0.28	0.32
Step 4			
Stage 4 end temperature (T_4_), °C	398	399	400
Weight change (m_4_), %	16.9	37.7	34.4
Temperature at V_max4_ (T_max4_), °C	372	373	374
Maximum degradation rate (V_max4_), %/°C	0.52	0.65	0.61
Step 5			
Stage 5 end temperature (T_5_), °C	550	550	550
Weight change (m_5_), %	23.3	16.5	12.1
Temperature at V_max5_ (T_max5_), °C	440	–	–
Maximum degradation rate (V_max5_), %/°C	0.28	–	–
Remainder at temperature 900 °C, %	25.2	23.0	19.7

**Table 5:** The results of plastograph analysis for PVC and PVC mixes with filler (RRGED).

Material	M_X_, Nm	T_X_, °C	t_X_, min	M_E_, Nm	T_E_, °C
PVC	37.6	180.4	14.1	31.5	184.7
PVC/20 RRGED	38.8	181.3	8.7	34.1	185.8
PVC/30 RRGED	39.6	183.8	8.6	33.9	189.7
PVC/40 RRGED	46.3	184.5	8.8	37.2	191.0

**Table 6:** (**a**) The results of thermal stability analysis carried out using the Congo red method and analysis of the TG and DTG curves within the range of 50 to 200 °C for the samples of PVC and PVC/RRGED composites. (**b**) Analysis results for the TG and DTG curves between 200 and 600 °C for PVC and PVC/RRGED composites.

**(a) Sample**	**S,** **min**	**Δm_165 °C_,** **%**	**T_max0P_, °C** **/V_max0P_, %/°C**	**Δm_0P_,** **%**		
PVC	21.8	0.1	-	0.2		
PVC/20 RRGED	29.5	0.7	163/0.011	1.0		
PVC/30 RRGED	30.6	0.9	160/0.015	1.2		
PVC/40 RRGED	31.4	1.1	165/0.026	1.7		
**(b) Sample**	**T_5%P_,** **°C**	**T_max1P_, °C** **/V_max1P_, %/°C**	**Δm_1P_,** **%**	**T_max2P_, °C** **/V_max2P_, %/°C**	**Δm_2P_,** **%**	**R_600P_,** **%**
PVC	276	310/1.57	61.4	478/0.40	26.1	12.1
PVC/20 RRGED	264	318/0.86	59.5	475/0.37	25.7	13.3
PVC/30 RRGED	262	304/0.81	57.0	472/0.32	24.0	18.5
PVC/40 RRGED	257	296/0.91	54.9	473/0.31	23.1	20.0

**Table 7:** Analysis of the DSC thermograms of PVC and PVC-based composites.

Sample	T_mP_, °C	ΔH_mP_, J/g	T_gP_, °C	ΔCp_P_, J/g °C
PVC	56.1	1.72	79.0	0.27
PVC/20 RRGED	55.3	1.04	76.0	0.27
PVC/30 RRGED	55.7	0.95	76.7	0.24
PVC/40 RRGED	54.5	0.53	76.3	0.21

**Table 8:** Test results of density, mechanical properties, hardness, and impact strength for PVC and PVC-based composites with RRGED.

Material	D, g/cm^3^	E_t,_ MPa	σ_y,_ MPa	ε_y,_ %	σ_m,_ MPa	ε_m,_ %	σ_b,_ MPa	ε_b,_ %	H, °ShD	U, kJ/m^2^
PVC	1.37 ± 0.01	1230 ± 90	55.3 ± 4.5	4.1 ± 0.4	55.3 ± 4.5	4.1 ± 0.4	34.2 ± 1.0	11.3 ± 1.1	65 ± 1	58.7 ± 5.5
PVC/20 RRGED	1.36 ± 0.01	1290 ± 70	–	–	29.7 ± 0.1	3.5 ± 0.2	26.9 ± 0.1	4.1 ± 0.2	66 ± 2	5.5 ± 0.5
PVC/30 RRGED	1.34 ± 0.01	1520 ± 10	–	–	23.6 ± 0.5	2.1 ± 0.2	22.8 ± 0.2	2.5 ± 0.3	67 ± 2	3.3 ± 0.1
PVC/40 RRGED	1.33 ± 0.01	1630 ± 10	–	–	21.2 ± 0.2	1.7 ± 0.3	20.8 ± 0.1	1.9 ± 0.3	68 ± 2	3.2 ± 0.5

**Table 9:** Flammability test results for PVC and PVC-based composites with RRGED.

Symbol	LOI, %	UL 94
PVC	37.4 ± 0.1	V0
PVC/20 RRGED	34.0 ± 0.1	V0
PVC/30 RRGED	32.4 ± 0.2	V0
PVC/40 RRGED	32.0 ± 0.1	V0

## Data Availability

Not applicable.
